# Reliability of Cone Beam Computed Tomography in Predicting Implant Treatment Outcomes in Edentulous Patients

**DOI:** 10.3390/diagnostics13172843

**Published:** 2023-09-02

**Authors:** Abdulaziz Alhossan, Yu-Cheng Chang, Tun-Jan Wang, Yu-Bo Wang, Joseph P. Fiorellini

**Affiliations:** 1 Department of Periodontics, School of Dental Medicine, University of Pennsylvania, Philadelphia, PA 19104, USA; tjwang@upenn.edu (T.-J.W.); jpf@upenn.edu (J.P.F.); 2 Department of Periodontics and Community Dentistry, Dental College, King Saud University, P.O. Box 60169, Riyadh 11545, Saudi Arabia; 3 School of Mathematical and Statistical Sciences, Clemson University, Clemson, SC 29634, USA; yubow@clemson.edu

**Keywords:** dental, implants, CBCT, predictability

## Abstract

Since the development of CBCT has been utilized in dentistry, the images of the CBCT can assist the surgeon to evaluate the anatomy carefully. Despite the value of radiology evaluation, implant procedures may require additional consideration rather than only evaluating the anatomical factors. The purpose of this study is to evaluate the predictability of using CBCT alone to plan for implant placement in edentulous patients digitally. CBCT images were analyzed by clinicians, measuring the maxillary and mandibular ridge heights and widths digitally of four predetermined implant sites in the maxillary and two selected implant sites in the mandibular arches of 91 patients planning for implant-supported overdenture. A total of 47 patients out of the 91 had completed implant placement on the edentulous ridge, contributing to 55 upper and/or lower arches (136 dental implants). Both predictabilities are low, implying that CBCT planning for implant placement on the edentulous ridge is not a good index and is insufficient to predict the surgical procedures as a solo method. The findings of this study indicate that digital planning by CBCT is insufficient to serve as an individual tool to predict implant procedures. Further information and evaluation must be considered for implant placement in the edentulous ridge.

## 1. Introduction

Individuals with poor oral hygiene have a higher prevalence of being edentulous and suffering from the inconvenience of daily activities [1,2,3]. The complete denture is one of the most popular treatment modalities to restore esthetics, phonetics, and function [4,5]. Complete denture was the standard method to fix the dentition. However, the stability and retention of the denture are always challenging due to alveolar bone loss over time. After the innovation of the implant-supported overdenture, the stability of the denture can significantly be improved [6].

For implant placement in the maxilla for a removable overdenture, a minimum of four implants are needed for a favorable result. As for the mandible, a minimum of two implants showed a favorable result, although placing four implants showed a slightly better outcome [7].

The morphology of the bone is considered one of the most crucial factors in implant placement and restoration [8]. Numerous anatomical and vital structures in the maxilla and the mandible affect and limit the treatment planning and the prosthesis of choice. The structures that affect the implant placement in the maxilla include: (1) the nasopalatine foramen, which transmits the palatine vessels and nasopalatine nerves; (2) maxillary sinus, which is a pyramid-shaped cavity bilaterally, and it is recommended to avoid membrane perforations. As for the anatomical structures of concern in the mandible, these include: (1) the inferior alveolar canal, which is a branch of the trigeminal nerve. The variation in the position of the canal makes implant placement challenging for the surgeon; (2) mental foramen and nerve, where the mental nerve emerges to supply innervation to the anterior mandible. The location of the mental foramen should be identified prior to implant placement to avoid injury to the nerve; (3) mandibular incisive canal, which is a continuation of the mandibular canal mesial to the mental foramen. The patient can feel pain or mild discomfort should they have large incisive nerve canals; and finally (4) lingual foramen and lateral canals, which are small vascular canals and commonly are in the midline and lateral to the midline. The injury of these minute canals may complicate surgery due to bleeding should larger canals exist [9]. To aid in detecting these structures and plan the implant procedure in advance, multiple applications can assist with diagnosis, surgical implant planning, and delivery of prostheses, such as a cone beam computed tomography (CBCT) [10,11]. CBCT imaging can generally evaluate the anatomy from multiple angles with reasonable radiation exposure. This is relatively important because the edentulous patients are missing the teeth that can be used as the reference, and it could be challenging to review the critical anatomical structure and plan for the implants [12,13,14].

Despite the CBCT being of important value in implant surgery, it has become a gold standard in the implant placement protocol. However, many clinicians may have forgotten that the success of implant surgery is not just the anatomy but also other factors such as the history of periodontitis, other systemic conditions, and the quality of the bone. Relying on CBCT alone to plan the surgical procedure may lead to unforeseen outcomes such as sinus perforation, buccal bonce dehiscence, or early implant failure. The aim of this study is to evaluate the predictability of using the CBCT alone to plan for implant placement.

## 2. Materials and Methods

This study is an investigator-initiated, retrospective clinical study that was approved by the institutional review board at the University of Pennsylvania. Clinical data, medical history, and cone beam computed tomography of edentulous patients were collected. All patients were planned to receive, and received the treatment of implant-supported overdenture in a dental school setting from 2015 to 2020 using an oral health database (AxiUm^®^, Software, Henry Schein, Melville, NY, USA). All patient information was collected without any identifiers, codes, links, or other means of associating the data to the subject’s identity. Demographic variables extracted included age, gender, race, smoking status, diabetes, and health status as classified by the American Society of Anesthesiologists (ASA I, II, III, or IV).

Three examiners performed the data collection and fabricated the implant treatment plan for implant-supported overdenture accordingly without reviewing the clinical records. Inclusion criteria consisted of any edentulous patient who received cone beam computed tomography and implant placements for implant-supported overdenture between 2015 and 2020. Exclusion criteria included dentate or partially dentate patients, implants within the edentulous arch and remaining roots, and patients with reported metabolic bone diseases.

**Figure 1:**
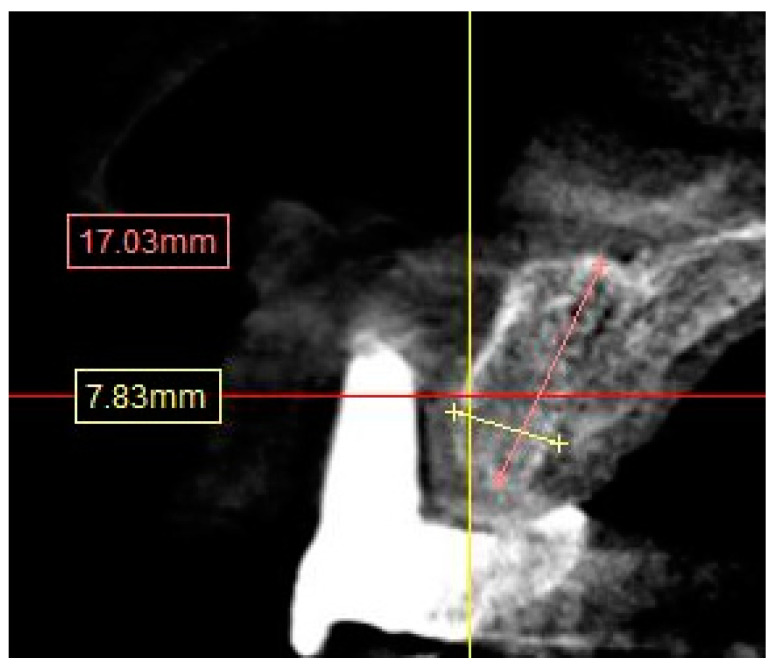
CBCT image showing height and width measurement calculation.

**Figure 2:**
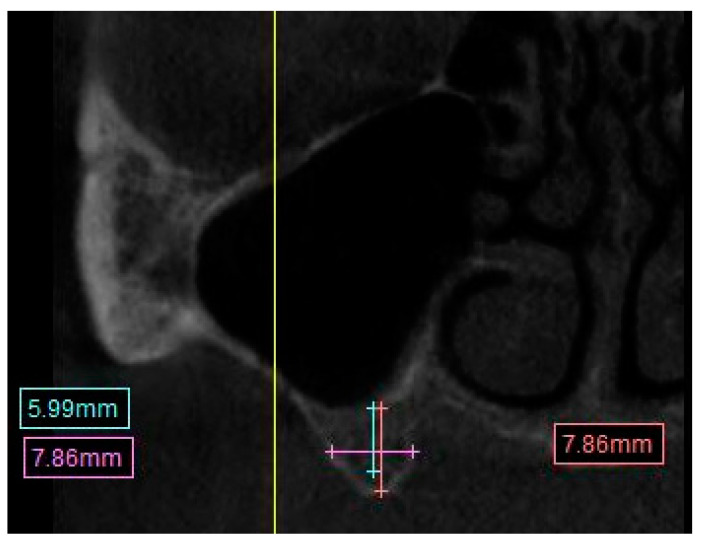
CBCT image showing measurement calculation for the distance from maxillary ridge crest to the floor of the sinus.

**Figure 3:**
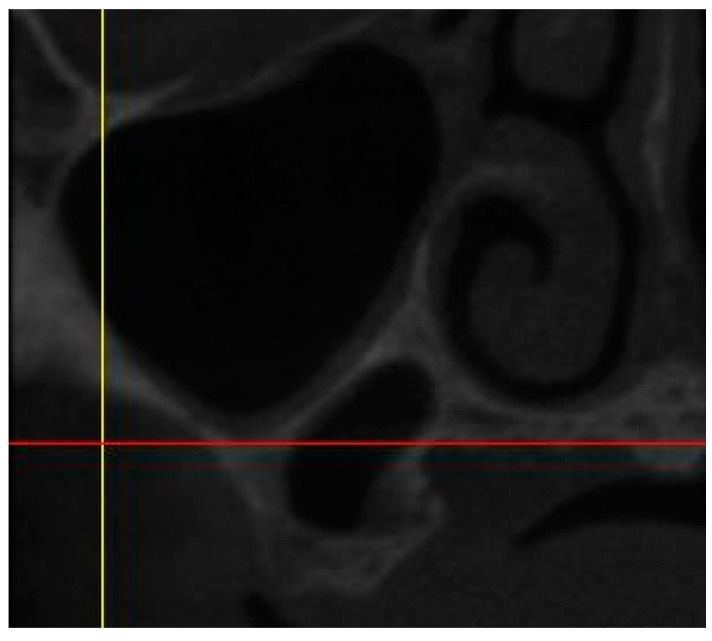
CBCT image showing the presence of sinus septum.

**Figure 4:**
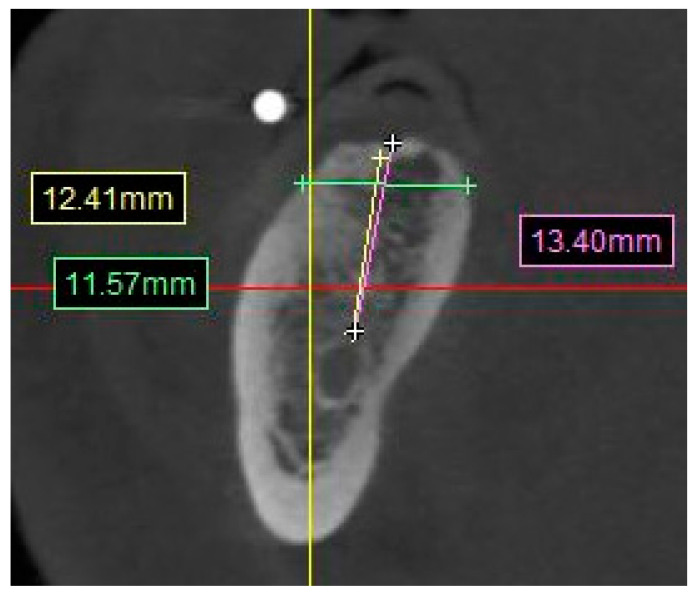
CBCT image showing the measurement calculation for the distance from mandibular ridge crest to the superior boarder of the inferior alveolar nerve canal.

**Figure 5:**
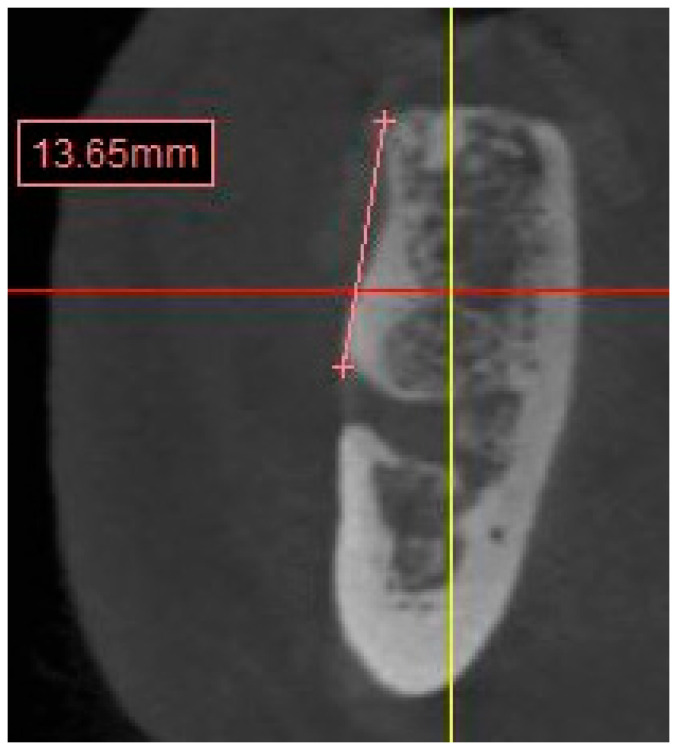
CBCT image showing the measurement calculation for the distance from mandibular ridge to the superior boarder of the mental foramen.

CBCT images were analyzed on Simplant^®^ (Dentsply Sirona, York, PA, USA). Images were reconstructed with slices of 1 mm thickness. A total of 12 sites on both maxilla and mandible, which represent the locations of the natural teeth, were measured to evaluate the anatomical structure, such as the width and height of the alveolar ridge and the distance to the anatomical landmarks (sinus floor, mental foramen, and inferior alveolar nerve) as follows:

- Residual anterior and posterior ridge heights and widths (maxillae and mandible) (Figure 1).
  ∘ Distances were calculated from the crest of the ridge. In the case of a thin ridge crest, alveoplasty was assumed and measurements were taken from the widest area that would accommodate the implant diameter. The alveoplasty was no more than 5 mm. The measurements were taken from the crest if more than 5 mm alveoplasty was needed, and the site was noted for grafting.
  ∘ The width was calculated 1–2 mm apically from the height start point.
- Distance from the crest of maxillary ridge to the floor of the maxillary sinus (Figure 2).
  ∘ A straight line from the crest of the planned molar location to the inferior border of the maxillary sinus.
- The presence of sinus septum, membrane thickening, and/or pathology (Figure 3).
  ∘ They were noted as yes/no.
- Distance from the posterior mandibular ridge to the inferior alveolar nerve (Figure 4).
  ∘ A straight line from the crest of the planned molar location to the superior boarder of the inferior alveolar nerve canal.
- Distance from crest of bone to the mental foramen (Figure 5).
  ∘ The measurement was taken from the most mesial slice onto which the mental foramen opens inside the oral cavity; the measurement was taken from the most superior boarder of the mental foramen to the edge of the crest in a straight line.

**Figure 6:**
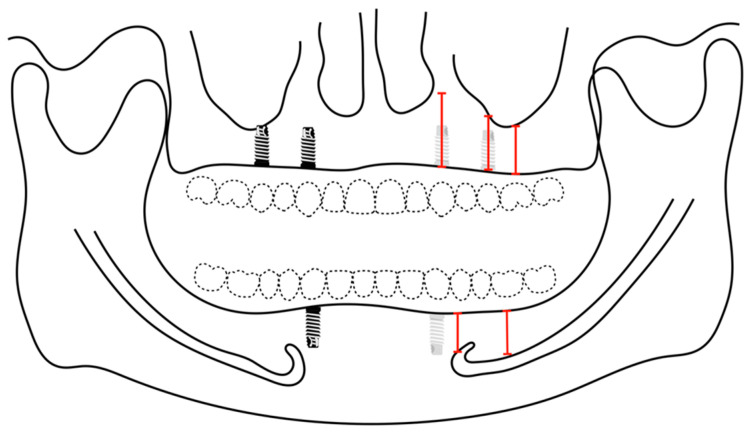
Digitally planned implant and anatomical analysis. Four implants (the area of lateral incisor canines, second premolars) are planned on the maxilla for implant-supported overdenture. Two implants (the area of canine) are planned on mandible for implant-supported overdenture.

The examiners had digitally measured the alveolar ridges of four implant sites in the maxilla and two implant sites in the mandible to place implants for an implant-supported removable prosthesis for both maxillary and mandibular arches (Figure 6). Four implants were planned for the removable prosthesis at the laterals or canines, and the second premolars areas for the maxilla. Two implants were planned at the area of the canine for the mandible. The surgical sites were investigated if they required additional augmentation procedures in advance or afterward, whether guided bone regeneration (GBR) or sinus elevation. Each site on the data collection sheet was given a number; the following numbers were considered and standardized when performing the digital planning:

1. Placing a standard-diameter implant size (4.1 mm).
2. Placing a narrow-diameter implant (3.3 mm).
3. The surgical site requires horizontal augmentation.
4. The surgical site requires vertical augmentation.
5. The surgical site requires vertical augmentation via internal sinus lift.
6. The surgical site requires horizontal and vertical augmentation.
7. The surgical site requires short implants (6 mm) or vertical augmentation with/or without horizontal augmentation.

After obtaining the measurements and digitally planning the implants, the individual charts were reviewed to correlate the treatment planning in the CBCT with the actual treatment rendered to the patient in the clinic.

To examine the predictability of digital planning, we considered CBCT planning as the covariate. At the same time, whether receiving the planned surgical procedure or not is the outcome of interest, we performed the mixed-effects logistic regression for all upper or lower implants to accommodate the situation where some patients have both upper and lower surgical procedures [15].

## 3. Results

A total of 222 patients were initially evaluated for inclusion in this study. A total of 131 patients were excluded for missing CBCT data and/or were partial dentated patients. A total of 91 patients’ CBCT files were utilized to digitally plan the implant placement with a mean age of 63.85 years old ±12.66. A total of 55 out of the 91 edentulous patients were males, which correlates to (60.4%) of the total sample, and 36 (39.6%) were females.

In regard to their health history, starting with the American Society of Anesthesiologists’ classifications, 5 patients out of the 91 (5.5%) were classified as ASA I, 60 (65.9%) were classified as ASA II, 25 (27.5%) were classified as ASA III, and 1 (1.1%) was classified as ASA IV. A total of 48 (52.7%) were smokers and 43 (47.3%) were non-smokers; regarding diabetes, 16 (17.6%) were diabetic and 75 (82.4%) were non-diabetic.

There were 75 maxillae and 55 mandibles for the 91 patients included in this study, of which 44 (48.6%) had their CBCT scans taken with radiographic guides during the scan.

**Figure 7:**
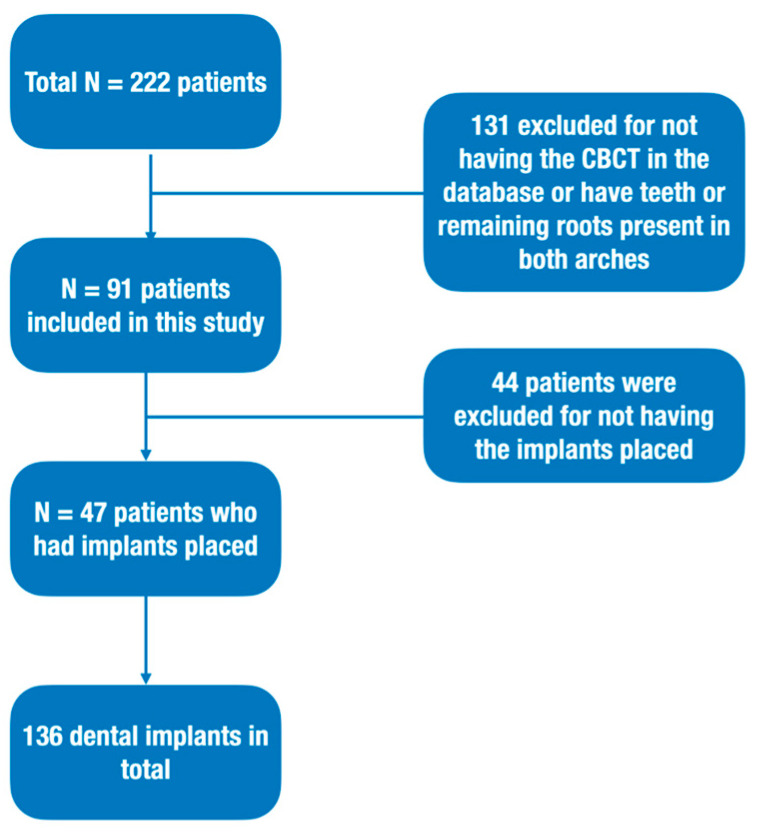
Study design workflow.

Another 44 patients were excluded because the implant procedure had not been carried out. Only 47 patients (55 arches) were included in the study to evaluate the predictability of predicting the implant through CBCT alone. (Figure 7).

From the total of 91 edentulous patients, the bone volume of the digitally planned implant sites was evaluated. Four implants were planned digitally on the maxilla second premolar and lateral incisor or canine area positions. The average height of the alveolar bone in the second premolar area is 8.89 mm and 6.78 mm in width. The average height of the canine area is 14.33 mm and 11.59 mm in width. The average height of the lateral incisor is 13.82 mm and 5.5 mm in width. Two mandibular implants were planned digitally on the lateral incisor or canine. The average height of the canine area is 13.7 mm and 7.49 mm in width (Table 1). The mean distance to the sinus floor is 5.66 mm. There are 31 (41.33%) sinus pathologies and/or thickening present, such as retentive cyst, and 16 (21.33%) sinus septa found in the total maxillae. A total of 18 (24%) of the second premolars were planned for lateral sinus lift, while 15 (10%) were planned for a vertical sinus lift. The mean distance to the mental foramen from alveolar crest bone is 9.08 mm, while, for the inferior alveolar nerve, the mean distance from the alveolar crest is 11.19 mm (Table 2).

### 3.1. Statistical Analysis

By using the *t*-test, the statistical differences between the cases that suggested with/without additional augmentation procedures for the placement of dental implant of the maxillary teeth are found in the continuous covariates: maxillary ridge height (*p*-value < 0.01), maxillary ridge width (*p*-value < 0.01), and distance to sinus floor right and left (*p*-value = 0.01 and <0.01, respectively), while age, sinus pathology, and sinus septum are insignificant. As for the discrete covariates, gender, ASA classification, smoking, and diabetes, no significant results are detected via the chi-square test. For the mandible, the significant differences between the cases that suggested with/without additional augmentation procedures for the mandibular implants are found in the mandibular ridge height (*p*-value < 0.01), mandibular ridge width (*p*-value < 0.01), distance to the mental foramen right and left (*p*-value = 0.01, *p*-value < 0.01, respectively), and distance to the inferior alveolar nerve right and left (*p*-value = 0.03, 0.02, respectively), while age, via the chi-square test, and the other demographic variables are not significant.

In the logistic regression model with LASSO regularizations for the maxilla on the suggested surgical procedures (bone augmentation or not) for the maxillary implants, only maxillary ridge height, maxillary ridge width, distance to the sinus floor left, and ASA classification (I & II vs. III & IV) are found to be significant and selected in the final chosen model. The result shows the expected changes of 0.26, 0.14, 0.02, and 0.45 in the log odds of no bone augmentation suggested by CBCT when there is one unit increment in the covariates (holding all the others constant), respectively. Meanwhile, for the mandible on the suggested surgical procedures (bone augmentation or not) for the lower implants, only lower ridge height, lower ridge width, and distance to the mental foramen left are detected with significant effects 0.29, 1.23, and 0.13 on the log odds of no bone augmentation suggested by the CBCT, respectively.

When evaluating the associations of the mean maxillary bone height with the demographic variables (age, gender, ASA classification, smoking, diabetes), only gender is shown with statistical significance (*p*-value = 0.01). Meanwhile, for the mean width, gender (*p*-value < 0.01) and diabetes (*p*-value = 0.03) are statistically significant. These results indicate that females tend to have smaller average maxillary teeth heights and widths than males, and the diabetes group tend to have higher average upper teeth widths than the non-DM patients in this study, while, for the mandible when evaluating the relationships between the mean mandibular bone height/width and the demographic variables (age, gender, ASA classification, smoking, diabetes), no significant results are disclosed.

### 3.2. Predictability of the CBCT Planning for Implant Surgery

In a total of 75 maxillae, in which implant-supported removable prostheses were treatment planned according to CBCT evaluation, 13 arches (17.33%) were able to have implants placed in second premolar areas, canines, or lateral incisor areas without any additional augmentation procedures carried out digitally. A total of 55 arches of mandible implant-supported removable prostheses were treatment planned according to CBCT evaluation, in which 35 arches (63.63%) were able to have implants placed in the canine position without any additional augmentation.

A chart review was conducted to compare the implant position and any need for additional augmentation procedures.

**Figure 8:**
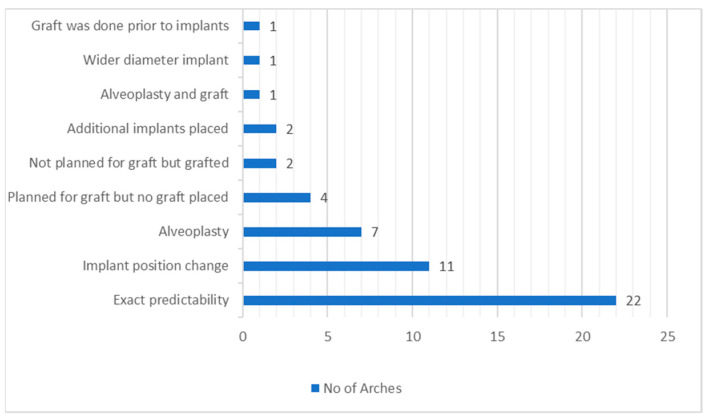
Predictability analysis for the 47 included patients.

From the 47 patients who had their digital planning compared with clinical treatment, we can appreciate the following variables (Figure 8):

- A total of 22 arches had exact predictability from digital planning.
- A total of 11 arches had the implant position changed.
- Seven arches had alveoplasty carried out, and one arch had alveoplasty and grafting carried out.
- Four arches were planned for grafting, but no graft was placed.
- Two arches were not planned for grafting but were grafted.
- Two arches had implants placed in the additional site rather than mandibular canines.
- One arch received a wider diameter implant than digitally planned.
- One arch had grafting carried out prior to implant placement surgery.

A total of 47 out of the 91 patients had completed the implant placement in the edentulous ridge clinically, contributing to 55 maxillary and/or mandibular arches. Based on the results of the mixed-effects logistic regression model, we observe that the probabilities of having the planned treatments of the digital planning are only 0.57 and 0.38, respectively, for cases that suggested with/without bone augmentation. It is clear that both predictabilities are low since a coin flipping already has a probability of 0.5, implying that CBCT planning for implant placement on the edentulous ridge is not a good index and is insufficient to predict the implant surgical procedures as a solo method. Further information is needed to guide the planning for future surgical procedures. (Table 3)

## 4. Discussion

In a review article by Orentlicher et al. [16], he stated that to successfully integrate the cone beam technology and the cone-beam-guided surgery, it must be acknowledged that a steep learning curve is required, and that dentists should pursue further continuing education to increase their understanding of the knowledge of CT scans, their digital and treatment planning software, and the digital workflow. Guerrero et al. [17] compared the alveolar grafting prediction between panoramic radiograph and CBCT images in 108 partially edentulous patients with 356 implants placed; they found that implant planning with cone beam computed tomography had a higher prediction and agreement of implant planning versus the panoramic-based surgery, and found that the sensitivity and the specificity of CBCT for implant complications were 96.5% and 90.5%, respectively, and, for the bone graft augmentation, 95.2% and 96.3%, respectively. Mello et al. [18] investigated the impact of CBCT on implant planning and prediction of the final implant size and concluded that cone beam computed tomography improved the prediction of the implant length and improved the accuracy in implant planning. Necking et al. [19] assessed the reliability of implant placement using a surgical guide after virtual planning with CT data and concluded that cone beam data and surgical guides could be reliable for preoperative planning of implant size, position, and anatomical complications.

The accuracy of CBCT in implant dentistry has also been extensively investigated. Al-Ekrish et al. [20] found that CBCT was associated with a clinically and statistically significant measurement error of 0.49 mm. A systematic review by Fokas et al. [21] concluded that CBCT can be considered as an appropriate diagnostic tool for planning, but a 2 mm safety margin is needed adjacent to anatomic structures. A systematic review by Anter et al. [22] indicated that the average CBCT measurement error ranged from 0.19 mm to 1.27 mm; they concluded that the evidence is not strong. Conversely, some studies showed that CBCT images underestimate the actual distances. A study by Lascala et al. [23] showed that the measurements were always larger than those for the CBCT images, but only significant for measurements of the internal structures of the skull base. Another study by Komuro et al. [24] showed that CBCT measurements were significantly smaller than model scanners, intra-oral scanners, and electronic caliper control.

Our study demonstrated that the mean maxillary posterior heights and widths were 9.35 mm and 6.87 mm, respectively, which meant that, on average, the posterior maxilla would require bone grafting for the placement of a standard diameter implant or the placement of a narrow diameter implant to avoid grafting. Similarly, the mean maxillary anterior heights and widths were 14.04 mm and 5.69 mm, respectively, which means that, on average, the anterior maxilla would require a grafting procedure for the placement of implants regardless of its size. As for the mandible, our study shows that the mean mandibular posterior heights and widths were 10.99 mm and 8.21 mm, respectively, which means that, on average, the posterior mandible, in contrast to the maxilla, could have implant placement with standard size implants without the need for secondary augmentation procedures. Similarly, the mean mandibular anterior heights and widths were 14.83 mm and 7.26 mm, respectively, which is also forgiving for placing dental implants without additional procedures. Compared with clinical treatment, some of the data in our study opted for alveoplasty to reduce the ridge height and gain width instead of using bone grafting as an option. In a similar study by Fiorellini et al. [25], where they used CBCT to evaluate bone availability for implant placement and sloped implant design, when they evaluated the ridge dimensions, they found out that in the posterior maxilla, the mean buccal bone height was 8.73 mm, the mean lingual bone height was 8.52 mm, and the mean width was 8.06 mm, while in the anterior area, the mean buccal bone height was 13.03 mm, the mean lingual bone height was 12.37 mm, and the mean width was 5.33 mm. As for the mandible, posteriorly, the mean alveolar buccal bone height was 10.18 mm, the mean alveolar buccal bone height was 11.01 mm, and the mean width was 7.49 mm; Anteriorly, the mean buccal bone height was 13.59 mm, the mean lingual height was 13.47 mm, and the mean width was 6.9 mm. These measurements were remarkably close to the measurements of this study; however, 60.6% of the site’s implants could be placed. This could be attributed to the placement of a single implant rather than four or two implants as a whole. A total of 39.4% of the sites were not adequate for implant placement, 56.5% of which needed additional guided bone regeneration procedures. Padhye et al. [26] evaluated 250 CBCTs with a total of 349 edentulous sites; they found that 55.45% of the molar and 54.42% of the premolar maxillary sites had a horizontal ridge width of less than 6 mm and concluded that additional augmentation procedures are required in a high percentage of the population in the posterior maxillary site when a standard-dimension implant is used.

In the present study, 43 of the maxillary molars (57.33%) and 18 of the maxillary second premolars (24%) required lateral window sinus lifts. In comparison, 26 of the maxillary molars (17.33%) and 15 (10%) of the maxillary second premolars required vertical lifts. Lan et al. [27] analyzed a total of 100 CBCTs. They concluded that a high percentage of edentulous sites in the posterior maxilla need sinus floor elevation for dental implant placement. Buser et al. [28] evaluated a total of 122 CBCT scans and found that the bone height decreased from premolar to molar areas, with first and second molar sites showing a bone height of less than 5 mm (54.12% and 44.64% respectively). Padhye et al. found that 67.83% of the molar and 44.86% of the premolar sites showed a height of less than 8 mm. Similarly, Fiorellini et al. found that, in 39.4% of the sites that were not adequate for implant placement, 43.5% required sinus augmentation procedures.

Suggestions for future studies would include having the same examiner place the dental implant to increase the accuracy, or prospectively treatment plan the cases for the residents.

## 5. Conclusions

This retrospective study evaluated the need for additional augmentation procedures for the placement of the dental implant for implant-supported removable prostheses and the predictability of digital planning alone on the clinical treatment as the primary objective. The low probability suggests that digital planning alone was not predictable, and other factors such as accuracy of the CBCT, bone density, and clinician error should be considered. Due to the limitation of this study and the small sample size, more data are needed in the future to confirm these findings.

## Figures and Tables

**Table 1:** Summary of bone volume on digitally planned implant locations.

Maxillary Teeth	Mean Height	Mean Width
Right Second Premolar	8.91 mm ± 5.67 mm	6.64 mm ± 2.17 mm
Right Canine	14.12 mm ± 4.01 mm	5.71 mm ± 1.61 mm
Right Lateral Incisor	13.88 mm ± 4.22 mm	5.48 mm ± 1.57 mm
Left Lateral Incisor	13.77 mm ± 4.29 mm	5.52 mm ± 1.66 mm
Left Canine	14.54 mm ± 3.76 mm	5.88 mm ± 1.71 mm
Left Second Premolar	8.87 mm ± 5.52 mm	6.93 mm ± 2.31 mm
**Mandibular Teeth**	**Mean Height**	**Mean Width**
Right Canine	14.32 mm ± 4.24 mm	7.68 mm ± 1.87 mm
Left Canine	13.09 mm ± 3.71 mm	7.31 mm ± 1.75 mm

**Table 2:** The average distance between the anatomical landmark and the bone crest.

Maxillary	Distance to
The right sinus floor	5.97 mm ± 4.35 mm
The left sinus floor	5.35 mm ± 3.69 mm
Mean	5.66 mm ± 0.43 mm
**Mandibular**	**Distance to**
The right mental foramen	9.07 mm ± 3.67 mm
The left mental foramen	9.08 mm ± 3.52 mm
Mean	9.08 mm ± 0.01 mm
The right inferior alveolar nerve	10.93 mm ± 4.09 mm
The left inferior alveolar nerve	11.46 mm ± 4.10 mm
Mean	11.19 mm ± 0.37 mm

**Table 3:** The predictability of the digital planned implant procedure.

Predictability	Augmentation Needed		Augmentation Not Needed	
As predicted	14		14	
Not as predicted	9		18	
**Mixed effects model**				
	Estimate	Std. Error	z value	Pr (>|z|)
(Intercept)	−0.49	0.48	−1.01	0.31
Bone augmentation suggested	0.75	0.63	1.20	0.23

## Data Availability

The data are not publicly available as per the IRB agreement from the University of Pennsylvania.
